# Detection and quantification of heavy metals and minerals in poultry feed collected from selected local markets in Bangladesh

**DOI:** 10.5455/javar.2024.k746

**Published:** 2024-03-12

**Authors:** Md. Khirul Islam, Sabbya Sachi, Quazi Forhad Quadir, Mahmudul Hasan Sikder, Muhammad Omar Faruque, Mohammad Alam Miah, Md. Shafiqul Islam, Arif Hossen Sani, Pollab Baishya, Kazi Rafiq

**Affiliations:** 1 Department of Pharmacology, Bangladesh Agricultural University, Mymensingh, Bangladesh; 2 Department of Agricultural Chemistry, Bangladesh Agricultural University, Mymensingh, Bangladesh; 3 Bangladesh Institute of Research and Training on Applied Nutrition (BIRTAN), Dhaka, Bangladesh; 4 Department of Physiology, Bangladesh Agricultural University, Mymensingh, Bangladesh; 5 Department of Agriculture Extension, Dhaka, Bangladesh; 6 Department of Livestock Services, Dhaka, Bangladesh; † These authors contributed equally

**Keywords:** Heavy metals, minerals, poultry feeds, spectrophotometry

## Abstract

**Objective:**

The study was primarily conducted to assess the stakeholders’ knowledge regarding the contamination caused by heavy metals in poultry feedstuffs. The concentration of some heavy metals (lead, chromium, cadmium, and nickel) and macro-minerals (sodium, potassium, and calcium) was also analyzed in poultry feeds collected from selected local markets in Sherpur district, Bangladesh.

**Materials and Methods:**

A well-structured questionnaire survey was used to investigate different stakeholders’ perspectives in relation to metal contamination in feed. Heavy metals and calcium were determined by atomic absorption spectrophotometry. The flame emission spectrophotometric technique was applied to determine sodium and potassium.

**Results:**

The majority of the stakeholders (90%) were found to have no knowledge regarding heavy metal contamination. Lead and nickel concentrations were below the detectable level in the collected samples. The average concentration of chromium in Jhenaigati upazila was four times higher than in Nalitabari upazila, at 21.806 mg kg^−1^ and 5.452 mg kg^−1^, respectively. The concentrations of cadmium in both brand and nonbrand samples exceeded the maximum allowable limit set by the European Union at 1.329 mg kg^−1^ and 1.328 mg kg^−1^, respectively. Sodium, potassium, and calcium were found in the ranges of 0.0011%–0.0035%, 0.0010%–0.0013%, and 0.0080%–0.0305%, which were extremely low in concentration compared to the minimum requirement in poultry feed.

**Conclusion:**

Regular surveillance and governance systems should be incorporated into national policy to cease the hazardous impacts of heavy metals through feed contamination. From a nutritional viewpoint, poultry feeds need to be critically formulated.

## Introduction

Since the last few decades, the risks associated with heavy metals from the perspective of food safety have been a great concern for public health worldwide [1]. As poultry-derived animal proteins are considered healthy, palatable, and economical [2], poultry farming is rapidly growing in Bangladesh to meet the increasing protein demand. The raw materials used for producing poultry feed are obtained from diversified sources. In Bangladesh, irrational disposal of tannery, ceramic, and textile wastes is mainly associated with heavy contamination of soil and vegetation [3]. Sometimes, wastes containing solid contaminants from tanneries are employed in feed production for protein sources in poultry [4].

The exposure of poultry feed to these anthropogenic sources of pollutants can contaminate the human food chain through various heavy metals [5]. Possessing the distinct properties of bioaccumulation, long biological half-lives, and nonbiodegradation in the living body, heavy metals can pose potential detrimental effects on the nervous, renal, reproductive, digestive, and cardiovascular systems of primary and secondary consumers, even if taken at minute concentrations for a longer period [6,7]. Any contamination during the formulation of poultry rations from individual raw materials ultimately gets associated with the consumer’s food chain [8]. Therefore, the finished products need to be explored to determine the potential sources and extent of heavy metal contamination.

According to the declaration of the US-EPA, lead (Pb), chromium (Cr), arsenic (As), cadmium (Cd), and nickel (Ni) are mostly responsible for environmental hazards [9]. Among the macrominerals, the dietary concentrations of sodium and potassium are crucial as they maintain acid–base balance and an optimal osmotic condition [10]. They also activate numerous intracellular enzymes and play important roles in neuromuscular conductivity [11]. Calcium is the most necessary element for bone mineralization. The dietary level of calcium should be critically considered to maintain the calcium and phosphorus ratio in poultry [12]. Any ignorance or lack of awareness among farmers, feed sellers, or feed manufacturers regarding the existence of heavy metals in feed and subsequent health risks may seriously hamper safe feed production. In Bangladesh, studies were conducted in industrial areas to investigate the presence of metals in chicken meat, eggs, and offals. Hossain et al. [13] found an excessive level of Pb, Cr, and Cd in the offals of poultry in Dhaka city, exceeding the maximum allowable limit of FAO/WHO.

Mottalib et al. [14] claimed that the chicken meat in Dhaka contained a higher extent of Cr, but the levels of Cd and Ni were within the allowable limit. The Pb content was found to exceed the recommended level in eggs collected from the commercial poultry farms in Dhaka [15]. As a potential source of heavy metal contamination through the food chain [16], poultry feeds have been investigated in some recent studies. Chowdhury et al. [17] concluded that the Pb content in poultry feeds surpassed the permissible limit in the Gazipur district. However, Hossain et al. [18] claimed that the Pb and Cd contents in commercial broiler feeds were within the acceptable range in Dhaka and Chittagong districts.

The holistic scenario of poultry feed origin heavy metal toxicity is still obscure in Bangladesh, and there is no data available for macro-mineral contents in feeds. Most of the research has been carried out in the city areas of Bangladesh, and there are intercity variations in heavy metal concentrations in poultry feeds [18]. Hence, emphasis should be given to observing the scenario of heavy metal contamination and mineral contents in poultry feeds in the agro-based regions of the country. As no data were found for the Sherpur district, the current work has been put into action with the objectives of investigating the stakeholders’ perspective on heavy metal contamination and analyzing poultry feeds for some selected heavy metals and macro-minerals.

## Materials and Methods

### Study area

**Figure 1.**
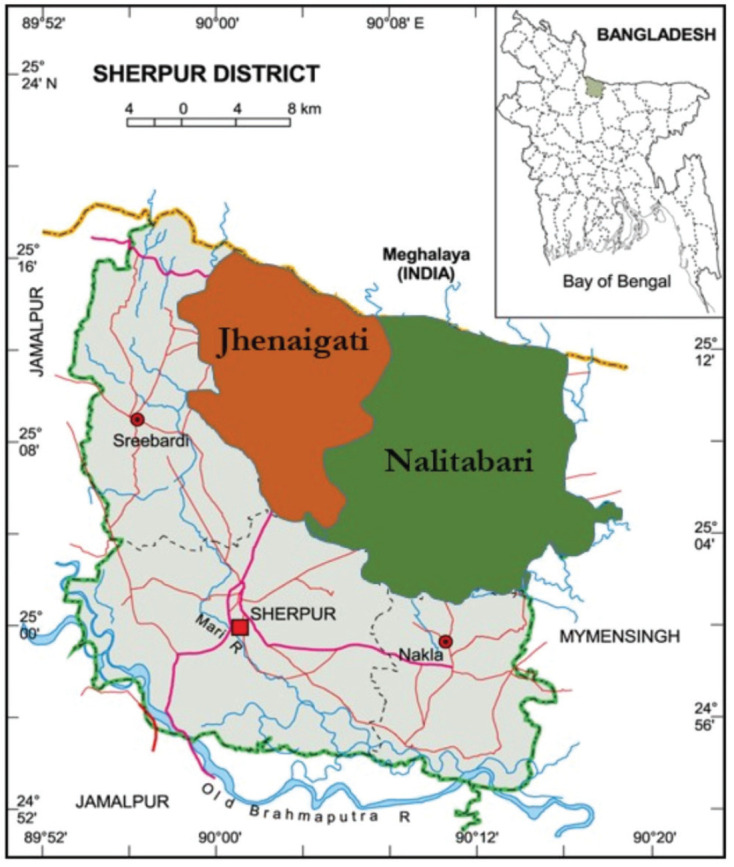
Map showing the sample collection area.

Two different upazilas of Sherpur district (Nalitabari and Jhenaigati) in Bangladesh were selected as sites for the collection of poultry feed samples (Fig. 1). Sherpur district is mainly an agro-based region in Bangladesh, covering an area of 1,363.76 square km with a population of around 1.5 million (Bangladesh National Portal, 2023; https://www.sherpur.gov.bd/en/site/page/CBvE) The study area comprises almost 40% of the district.

### Questionnaire survey

We designed a semi-structured questionnaire to survey farm owners and shopkeepers. The main aim of collecting the data was to assess and correlate the knowledge, awareness, and educational status of the stakeholders regarding heavy metal contamination. We used both English and Bengali in making the questionnaire to improve the output. Moreover, a short-term training program was arranged for the investigators to facilitate their communication with the respondents effectively and obtain actual information from the questionnaire.

### Collection of samples

Feed samples (10 brand and 12 nonbrand samples) were collected randomly from the selected study areas. An amount of 250 gm sample was collected in each case of sampling and kept in an airtight plastic zipper bag labeled with a separate identical code. The Department of Pharmacology prepared the samples for subsequent analysis at the Department of Agricultural Chemistry of Bangladesh Agricultural University.

### Sample preparation

First, the samples were dried for 48 h in an oven maintained at 60°C to obtain a constant dry weight. The dry products thus obtained were ground and mixed with the help of a mortar and pestle for homogenization, which were stored in individual plastic zipper bags until further processing.

### Sample digestion

The procedure described by Islam et al. [16] was followed with a few modifications for the digestion process. We mixed 1 gm of each sample with a 10 ml solution of perchloric acid and nitric acid (1:2) in a conical flask. 2 ml of hydrogen peroxide solution was added to it and heated at 150°C until a colorless digest was obtained, which was followed by cooling and filtering using the Whatman No. 42 filter paper. The digest volume was adjusted by adding deionized water to make it 50 ml. The final volume of the digest was poured into an airtight plastic bottle to store it in freezing conditions with proper labeling for future analysis.

### Analysis of heavy metals

An atomic absorption spectrophotometer (AAS) (SHIMADZU, AA-7000, Japan) was employed to determine the selected metals. According to the standard protocol provided by the manufacturer, the operational condition of the AAS was maintained, and the machine was calibrated. The absorbance reading for each standard solution of metal was plotted against the known concentration to produce a standard curve. The level of metals in a sample was determined by evaluating the AAS reading and calibration curve. A blank solution of deionized water was also employed to verify if there were any inaccuracies in the standards’ reading, which was followed by making the necessary corrections.

### Determination of macro-minerals

The concentrations of sodium and potassium in poultry feed were determined separately with the help of a flame emission spectrophotometer (Model Jenway PET 7) using appropriate filters. About 50 ml of the filtered sample was taken in a 100-ml beaker in each case and aspirated into a gas flame set at 10-PSI air pressure. The absorption wavelengths for sodium and potassium were 587 and 768 nm, respectively. The intensity of light emission was recorded in percentages to determine the concentrations of the minerals. The content of calcium was determined by following a similar protocol as described for heavy metals.

### Data compilation and processing

Data compilation and statistical analysis were performed using the software Statistical Package for Social Sciences (SPSS) (IBM SPSS, version 22). The results were expressed as mean ( ± SD), and hypothetical analysis was performed by using the level of significance at 0.05 and 0.01.

## Results

**Figure 2.**
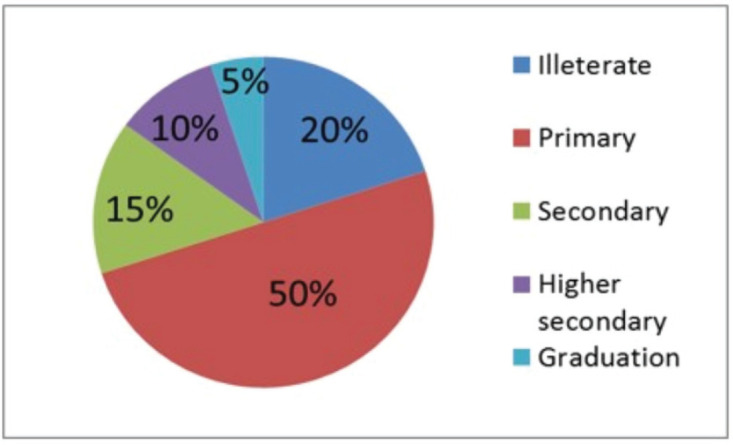
Educational qualification of farmers and feed sellers.

**Figure 3.**
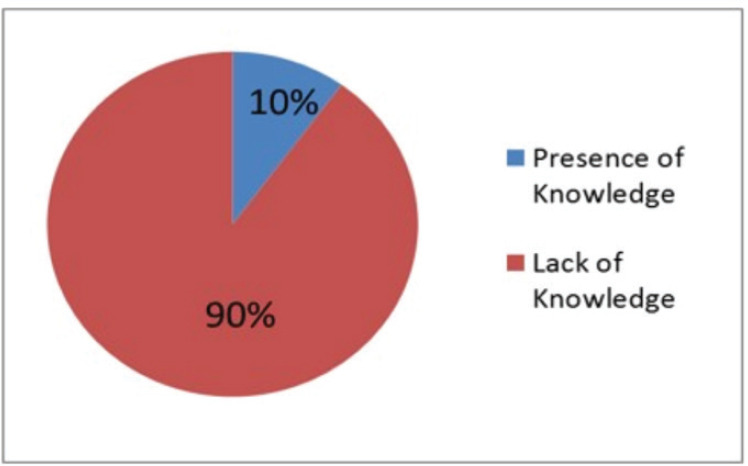
Knowledge and awareness of farmers and feed sellers regarding heavy metal contamination.

On the basis of the questionnaire survey, half of the respondents (farmers and feed sellers) were found to have completed their primary level of education, and the least number, 5%, were graduates (Fig. 2). The finding regarding knowledge and attitude was alarming, as 90% of the respondents did not know about heavy metal contamination (Fig. 3).

### Content of heavy metals

Lead and nickel concentrations were observed below the detectable level in all the samples. The quantification results of the selected metals are presented in Table 1. The mean ( ± SD) concentrations of Cr in Nalitabari and Jhenaigati were 5.452 ± 3.403 and 21.806 ± 6.087 mg kg^−1^, respectively. In the case of Cd, the mean ( ± SD) values found in Nalitabari and Jhenaigati upazila were 1.362 ± 0.252 and 1.296 ± 0.167 mg kg^−1^, respectively. Although Cr concentrations in Nalitabari and Jhenaigati differed significantly (*p* < 0.001), the variation of Cd in the upazilas was statistically insignificant (*p* > 0.05). Overall, the brand feed samples contained a comparatively lower level of Cr, 9.868 ± 7.372 mg kg^−1^ as compared with the nonbrand samples (16.763 ± 10.484 mg kg^−1^. The mean Cd concentrations were almost the same in the brand and nonbrand samples, at 1.329 ± 0.268 and 1.328 ± 0.163 mg kg^−1^, respectively. A significant difference was not revealed in the cases of brand and nonbrand samples of both Cr and Cd (*p* > 0.05).

**Figure 4.**
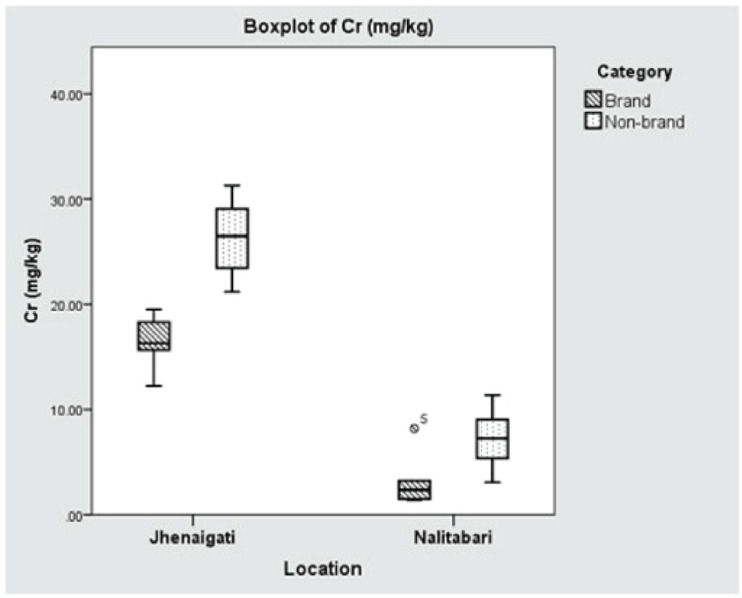
Boxplot showing location (upazila) and manufacturer wise concentration of Cr (mg kg^−1^). Horizontal lines in the box denote the median values. Box whiskers indicate the ranges of data values.

**Figure 5.**
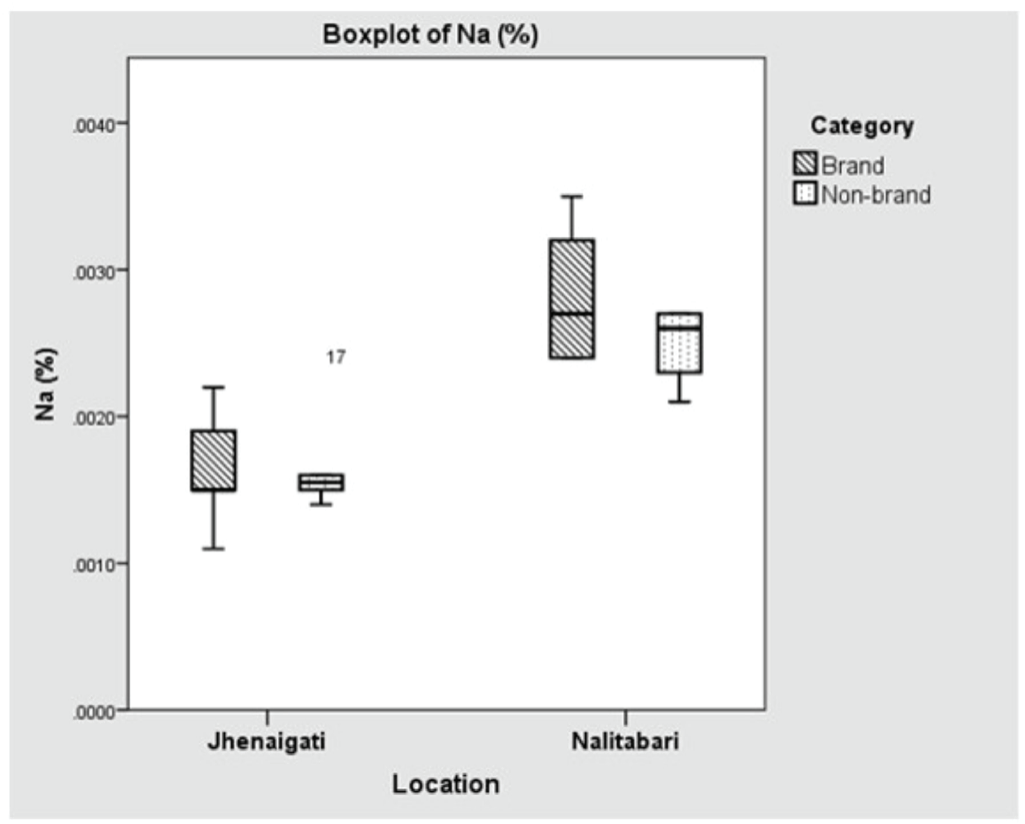
Boxplot showing location (upazila) and manufacturer wise concentration of Cd (mg kg^−1^). Horizontal lines in the box denote the median values. Box whiskers indicate the ranges of data values.

The manufacturer-wise concentrations of Cr and Cd in the selected regions are demonstrated in Figures 4 and 5, respectively. Though the brand feeds collected from Nalitabari and Jhenaigati upazila were from the same companies’ different lots, it had been a special concern if there were any significant differences in findings between the two different lots. The maximum concentration of Cr, 16.396 mg kg^−1^ was detected in brand feeds collected from Jhenaigati upazila in a range of 12.22 to 19.52 mg kg^−1^, while in Nalitabari, the brand feeds contained 3.34 mg kg^−1^ Cr with a range of 1.41 to 8.19 mg kg^−1^. The concentration of Cd found in brand feeds of Nalitabari was 1.366 mg kg^−1^ (1.05–1.83 mg/kg), while in Jhenaigati Upazilla, the value was 1.292 mg kg^−1^ (1.02–1.53 mg kg^-1^). A significant statistical difference was detected (*p* < 0.05) in the case of Cr content between two different lots of the same company.

### Content of minerals

Na, K, and Ca contents in the feeds were recorded in the ranges of 0.0011% to 0.0035%, 0.0011% to 0.0013%, and 0.0144% to 0.0273%, respectively. The mean ( ± SD) concentrations of the minerals in the feed samples according to the regions and manufacturers are shown.

**Figure 6.**
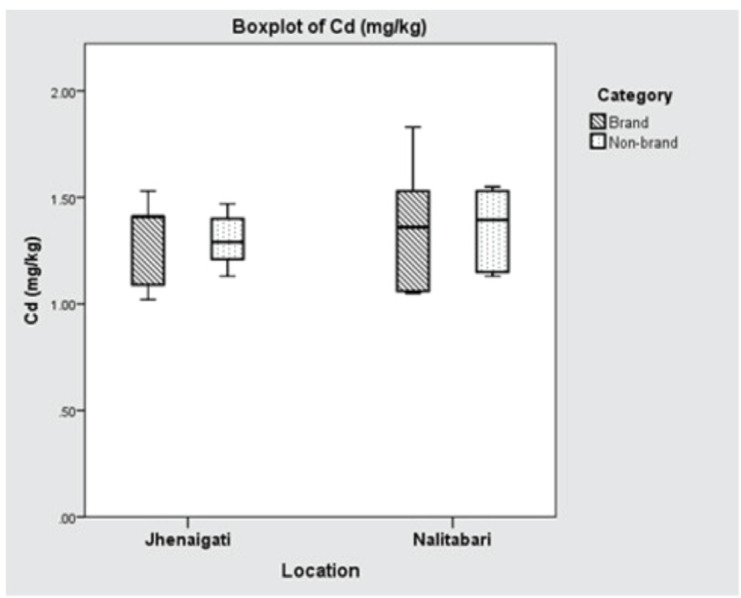
Boxplot showing location (upazila) and manufacturer wise concentration of Na (%). Horizontal lines in the box denote the median values. Box whiskers indicate the ranges of data values.

**Figure 7.**
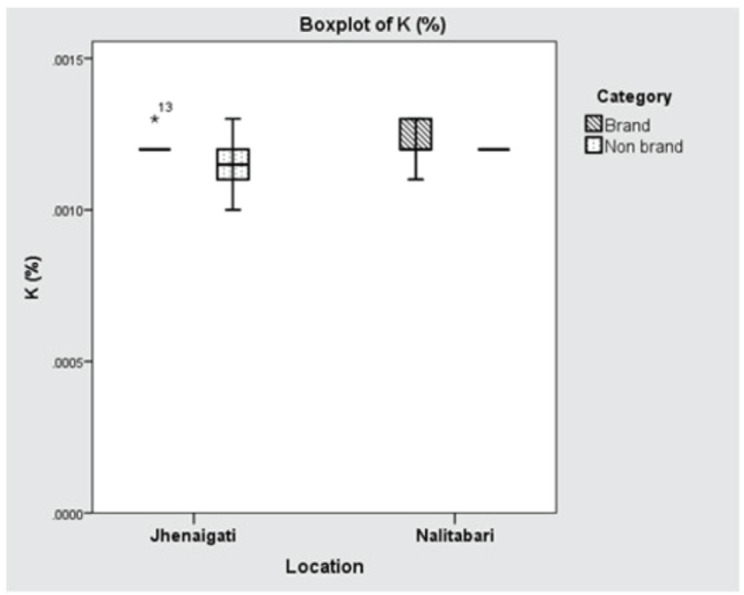
Boxplot showing location (upazila) and manufacturer wise concentration of K (%). Horizontal lines in the box denote the median values. Box whiskers indicate the ranges of data values.

**Figure 8.**
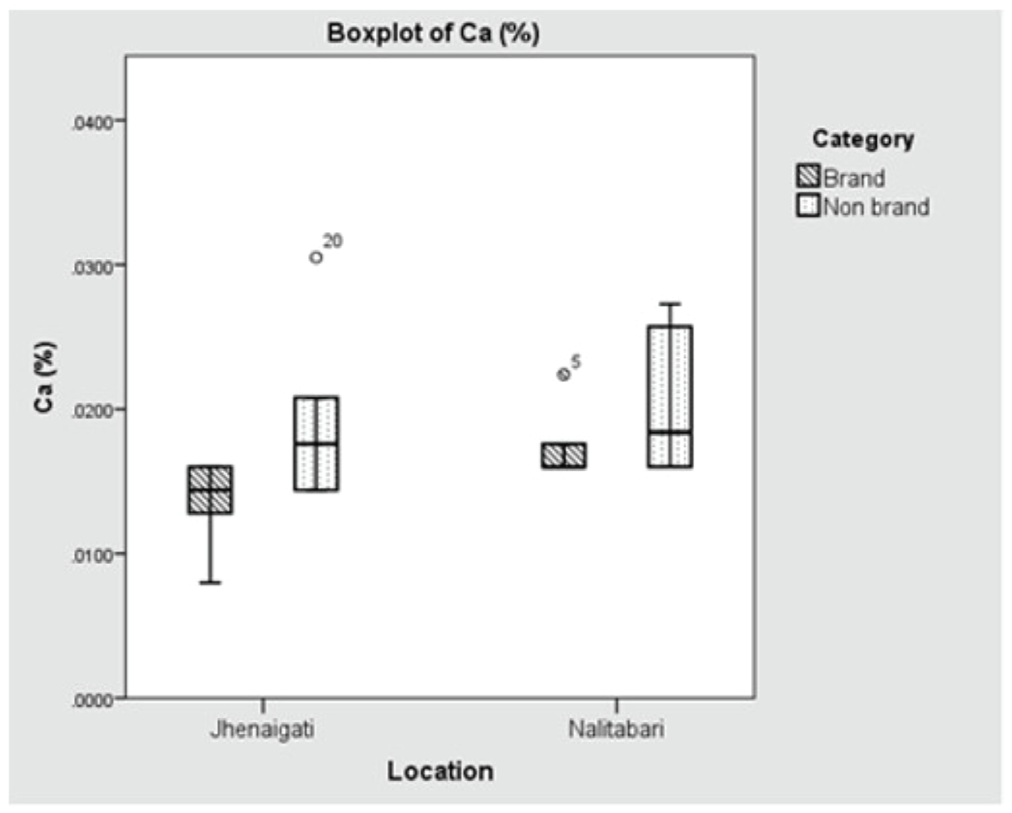
Boxplot showing location (upazila) and manufacturer wise concentration of Ca (%). Horizontal lines in the box denote the median values. Box whiskers indicate the ranges of data values.

The concentrations of Na in Nalitabari and Jhenaigati were 0.00266% and 0.00165%, respectively, which was found to have significant variation (*p* < 0.001) between the upazilas. The average content of K in Nalitabari and Jhenaigati were almost the same, at 0.00121% and 0.00118%, respectively, the difference of which was not statistically significant (*p* > 0.05). Although Ca contents in Nalitabari and Jhenaigati were 0.01907% and 0.01659%, respectively, statistical analysis showed the variation was statistically insignificant (*p* > 0.05). The mean difference of Na content in both the brand and nonbrand feed samples was potentially significant (*p* < 0.001), while in the cases of K and Ca, the concentrations did not vary significantly (*p* > 0.05). The concentration of macro-minerals (Na, K, Ca) in brand and nonbrand samples of the study area are shown in Table-2. The manufacturer-wise existence of Na, K, and Ca in the selected regions is displayed in Figures 6–8**,** respectively.

## Discussion

The questionnaire survey suggests that about 15% of the people are illiterate, and almost 90% of the people in the selected upazilas did not have any knowledge regarding heavy metal contamination. Illiteracy and a lack of knowledge may be the causes of using contaminated and substandard feeds for poultry production. Necessary steps should include increasing mineral concentration and decreasing heavy metal contamination in poultry feeds.

**Table 1.** Concentration of heavy metals in brand and nonbrand samples of the study area.

Metals	Location	Mean ± SD (mg/kg)	*p*-value	Manufacturer	Mean ± SD (mg/kg)	*p*-value	Reference standards (mg/kg)
Pb	Nalitabari	BDL	—	Brand	BDL	—	5
	Jhenaigati	BDL		Non-brand	BDL		
Cr	Nalitabari	5.452 ± 3.403	0.000	Brand	9.868 ± 7.372	0.087	500
	Jhenaigati	21.806 ± 6.087		Non-brand	16.763 ± 10.484		
Cd	Nalitabari	1.362 ± 0.252	0.476	Brand	1.329 ± 0.268	0.994	0.5
	Jhenaigati	1.296 ± 0.167		Non-brand	1.328 ± 0.163		
Ni	Nalitabari	BDL	—	Brand	BDL	—	0.3
	Jhenaigati	BDL		Non-brand	BDL		

BDL = Below detectable level in mg/kg and SD = Standard deviation.

In the regulations of the European Union (EU), the maximum permissible limit set for Pb is 5 mg kg^−1^ [19]. Jothi et al. [20] revealed the presence of Pb in feeds collected from all the districts of Bangladesh within the range of 7.37–52.25 mg kg^−1^. Pb content in the samples from Nalitabari and Jhenaigati upazila had been found below the detectable level. Both the brand and nonbrand samples did not contain any detectable levels of Pb. This is a good sign, which denotes that heavy metal contamination is decreasing in the Sherpur district. It can also be considered an indicator of the aspects of Pb contamination for similar types of agro-based rural regions in Bangladesh. This result suggests that manufacturing companies are using raw materials that are free from Pb contamination. Moreover, the upazilas are free from tannery, ceramic, and textile waste contamination, as in the cases of industrial areas in Bangladesh. This can be attributed to the absence of detectable Pb in the nonbrand samples. This might also be due to the variation in feed composition and collection sites for sample collection.

The maximum allowable concentration of Cr in poultry feed stipulated by the National Research Council (NRC) is 500 mg kg^−1^ [21]. The current investigation revealed the Cr concentration in a range of 5.452 ± 3.403 to 21.806 ± 6.087 mg kg^−1^. Although the findings surpassed the previous study outcome at Sadar Upazila in Mymensingh district [22], the concentration was much lower than the maximum allowable limit. The nonbrand samples contained a higher level of Cr as compared with the brand feed samples. Brand feed companies may be more conscious about using raw materials of good quality to avoid heavy metal contamination in feed. This may be due to variations in the sample collection site and differences in raw materials.

The estimated value of Cd in the study was more than 2.5 times higher than the recommended level of 0.5 mg kg^−1^ set by the EU in 2013 [19]. Islam et al. [23] found the Cd level in poultry feed ranged between 1.167 and 2.093 mg kg^−1^ in Mymensingh district, which is almost similar to the Cd content of feed collected from Nalitabari and Jhenaigati upazila, which ranged from 1.05 to 1.83 and 1.02–1.53mg kg^−1^, respectively. The raw materials might have contained a higher level of Cd, which could be responsible for the increased Cd content in feeds. The soil of the area from which raw materials were collected might contain increased Cd. The usage of tannery wastes as a protein source of poultry feed in certain cases, may conduce to the increased level of Cd. The mean Cd concentration in both the brand feed and nonbrand feed exceeded the permissible limit set by the EU.

Nickel is not normally added to chicken rations. However, the existence of Ni in poultry feeds has been evidenced by Kabir et al. [24] in a range of 4.40 to 23.00 mg/kg in some selected regions of Bangladesh. Here, all the samples collected from Nalitabari and Jhenaigati upazila did not contain any detectable levels of Ni. This result suggests that feed-producing companies are trying to produce qualified poultry feed free from heavy metal contamination. Overall, the variation of the sampling area, feed composition, ingredient quality, anti-nutritive factors, or possible technical errors might play roles in influencing the heavy metal content and detection in compound feeds, as reported in previous investigations [25].

Both the brand and nonbrand feed samples collected from the upazilas could not meet the minimum dietary requirements for Na, K, and Ca. Concentrations of Na in feeds collected from Nalitabari, 0.0026%, and Jhenaigati, 0.0016%, were much lower than the minimum dietary level, 0.12%–0.2% for broilers, recommended by the NRC [26]. In the present investigation, the lower level of Na detected in feed samples might be due to the raw materials used. Feed-producing companies might not be concerned enough about adding an appropriate amount of Na to poultry rations.

**Table 2.** Concentration of macro-minerals in brand and nonbrand samples of the study area.

Minerals (%)	Location	Mean ± SD	*p*-value	Company	Mean ± SD	*p*-value	Reference standards
Na	Nalitabari	0.00266 ± 0.00039	0.000	Brand	0.00224 ± 0.00077	0.556	0.12%–2% [23]
	Jhenaigati	0.00165 ± 0.00035		Non-brand	0.00208 ± 0.00052		
K	Nalitabari	0.00121 ± 0.00005	0.389	Brand	0.00122 ± 0.00006	0.150	0.4%–0.6% [24]
	Jhenaigati	0.00118 ± 0.00009		Non-brand	0.00118 ± 0.00008		
Ca	Nalitabari	0.01907 ± 0.00429	0.261	Brand	0.01552 ± 0.00362	0.048	1% [23]
	Jhenaigati	0.01659 ± 0.00587		Non-brand	0.01976 ± 0.00543		

SD = Standard deviation.

Ca contents were 0.0191% and 0.0166% in the cases of Nalitabari and Jhenaigati, respectively, which were much lower than the recommendation level of 1% as suggested by the NRC [26]. Here, decreased Ca concentration might be due to the location of the sample collection area and not adding calcium properly as a ration in poultry feed. Raw materials might have also contained a decreased level of calcium or possible technical errors in the investigation process.

Puls [27] stated that the optimum level of K in the feed mix of poultry should range in a range of 0.4%–0.6%, and 0.1% is regarded as K deficient. The concentration of K was recorded at 0.0012% for both upazilas, which was much lower than the recommendation level of 0.4%–0.6%. Here, the decreased K concentration might be due to the raw materials of the samples that were used for the preparation of food. Any other chemicals in the sample may also cause a variation in the result.

The limited sample size and the small study area may be considered shortcomings of the study. More extensive research is needed in Bangladesh to identify the critical control points of heavy metal contamination and to determine measures for minimizing health risks.

## Conclusion

The existence of heavy metals in poultry feed beyond the maximum allowable limit has been a serious threat from a human health perspective. Although lead and nickel concentrations were found below the detectable level and chromium was within the safety range, the concentration of cadmium in feed surpassed the upper margin of the permissible range. In addition, the concentration of all the minerals (sodium, potassium, and calcium) was found to be extremely low in feeds compared to the standard requirement. The inadequate amount of minerals and excessive amounts of heavy metals in feed have direct deleterious effects on poultry health and production performance and cause subsequent economic losses. Therefore, continuous monitoring and governance systems should be incorporated into the policy to ensure the standard quality of poultry feed.

## List of abbreviations

AAS, Atomic absorption spectrophotometer; BDL, Below detectable level; EU, European Union; NRC, National Research Council; SD, Standard deviation; SPSS, Statistical Package for Social Sciences; US-EPA, United States Environmental Protection Agency.
